# Environmental triggers of pemphigus vulgaris and bullous pemphigoid: a case control study

**DOI:** 10.3389/fmed.2024.1441369

**Published:** 2024-10-22

**Authors:** Corey Stone, Grace Bak, Daniel Oh, Cathy Zhao, Supriya Venugopal, Kuldeep Kumar, Dedee F. Murrell

**Affiliations:** ^1^Department of Dermatology, St George Hospital, Sydney, NSW, Australia; ^2^Faculty of Business, Bond University, Gold Coast, QLD, Australia; ^3^Faculty of Medicine, University of New South Wales (UNSW), Sydney, NSW, Australia

**Keywords:** autoimmune blistering disease, bullous disease, pemphigus, pemphigoid, epidemiology, case–control

## Abstract

**Background:**

Previous case–control studies have suggested that environmental factors including exposure to pesticides and organic materials, diet and medications have an important role in the pathogenesis of pemphigus vulgaris. These studies lacked geographical population controls and had less than three controls per case.

**Objective:**

To identify environmental and occupational risk factors associated with the development of pemphigus vulgaris (PV) and bullous pemphigoid (BP).

**Method:**

Cases were patients with PV (*n* = 25) and BP (*n* = 29) recruited from 2009 to 2017. Controls for PV (*n* = 72) and BP (*n* = 84) were recruited from the general population via electoral commission matching, matched for age, sex, residential location, and ethnicity. Data about demographics, environmental exposures and occupational exposures, was collected using a structured questionnaire. Conditional logistic regression analysis was undertaken using SPSS software to identify significant variables.

**Results:**

Significant factors associated with PV included the daily consumption of leeks (odds ratio (OR) 3.6; *p* = 0.025), mustard oil (OR = 4.4; *p* = 0.049), tomatoes (OR = 4.735; *p* = 0.032), multivitamins (OR 3.6; *p* = 0.009), alcohol (0.039), and calcium supplements (OR = 44, *p* < 0.001). Other associated factors included the number of lifetime sunburns (*p* = 0.019), high levels of mental stress (*p* < 0.001), and the use of lime household cleaning products (*p* < 0.001), Significant factors associated with BP included the daily consumption of green or herbal tea (OR = 3.7; *p* = 0.004), fish oil (OR = 5.7; *p* < 0.001), calcium supplements (OR = 6.1; *p* < 0.001), multivitamins (OR = 2.6; *p* = 0.043), and glucosamine (OR = 3.0; *p* = 0.046). The use of lime household cleaning products (*p* < 0.001) and high levels of mental stress (*p* = 0.007) were also associated with BP.

**Conclusion:**

Dietary factors containing thiol groups such as leeks, tomatoes, and mustard oil may be potential triggers for PV. High levels of mental stress, the use of supplementary medications such as calcium and multivitamins, and chemical cleaning products containing lime may be associated with an increased risk of developing both PV and BP. Lifestyle changes should be part of routine management for these patients.

## Introduction

Autoimmune blistering disease (AIBD) are a rare group of chronic and potentially life-threatening skin and mucosal diseases caused by autoantibodies targeting skin adhesion proteins (1). Among AIBDs the most common are bullous pemphigoid (BP), caused by autoantibodies targeted at hemidesmosomes in the dermal-epidermal junction and resulting in subepidermal blistering, pemphigus vulgaris (PV), and pemphigus foliaceous (PF) which are triggered by autoantibodies targeting intraepithelial desmosomes and resulting in intraepidermal blistering.

Although genetic predispositions have been identified in both bullous pemphigoid and pemphigus, environmental factors in the pathogenesis of AIBDs is yet to be established despite playing an inexplicably important role in the initiation and exacerbation of the disease process.

Pemphigus is caused by antibody mediated (type II) hypersensitivity reactions where IgG antibodies bind to intracellular desmosomal proteins desmoglein1 and desmoglein3. This binding induces disruption of the intercellular adhesive function of desmogleins leading to separation of neighbouring keratinocytes, a process referred to as acantholysis (loss of coherence between cells). Clinically, PV is characterised by thin-walled flaccid blisters, containing transudate fluid, which easily rupture leading to pruritic and painful erosions.

Despite being observed worldwide, pemphigus vulgaris has varied prevalence in different ethnic groups with a worldwide incidence of 0.1 to 0.5 per 100,000 population. In Finland the incidence is approximately 0.76 new cases per million per year whereas in Israel, rates are reported to be 16.1 per million per year (2). This is attributable to increased prevalence of several distinct HLA class II genes, specifically HLA-DRB1*04, HLA-A*10, and a genomic segment on 8q11.23 which spans ST18 gene (3, 4). Pemphigus prevalence in men and women is approximately equal with the average age of onset between the fourth and sixth decade, with rare cases occurring in pediatric populations. However, genetic predisposition alone is insufficient to lead to the development of the disease as an inciting trigger is often identified.

Numerous case–control studies of pemphigus patients have attempted to implicate a variety of environmental and occupational factors as triggers for PV disease onset. Studies to date have focussed on a wide range of potential triggers including organophosphate pesticides, heavy metals, medications, ultraviolet (UV) exposure, viral infections, emotional stress, and tobacco use (5–9). Suspected dietary factors include thiol compounds (garlic, leek, chives, onion, shallot), phenols (black pepper, red chillies, mango, cashew), tannins (tea, red wine, spices, raspberry, cranberry, blackberry), isothiocyanates (mustard, horseradish, cauliflower), and phycocyanins (Spirulina platensis alga) (10–15).

Despite some environmental exposures being linked to initiation of disease, all studies were suboptimal in design with only 1–2 controls per case and controls being recruited from dermatology clinics, i.e., patients with existing dermatological disease rather than three to four controls per case matched for geographical residence.

Bullous pemphigoid (BP) is the most common autoimmune skin disease of the pemphigoid group, characterised by autoantibodies against structural proteins of the hemidesmosomes, BP180 and BP230 (16). The clinical manifestations of BP involve tense subepidermal blisters forming on urticarial plaques, erythematous, or normal skin with associated pruritus (17). The presence of a precipitating trigger is identifiable in less than 15% of BP patients which suggests that environmental factors may not contribute as strongly to the development of BP as in PV (18). To date the strongest association with disease is the presence of other comorbidities such as cerebrovascular incident, or a neurodegenerative disease (19, 20). Numerous case–control studies have found that BP patients were more likely to have a history of neurological disease, and it is hypothesised that the molecular mimicry between the similar epithelial and neuronal isoforms of the BP antigens is attributable to this association (21, 22).

A number of medications have also been linked to increased risk of BP including dipeptidyl peptidase IV (DPP IV) inhibitors, neuroleptics, and diuretics (23–25). A proposed mechanism by which drugs may alter the antigenic properties of the skin is through binding to molecules within the lamina lucida of the basement membrane zone (BMZ), thereby acting as neoantigens and triggering the formation of anti-BMZ antibodies (26, 27). Similarly, a number of vaccinations have been postulated to induce or unmask subclinical BP by triggering a non-specific immune response in individuals with an immunologic predisposition (28).

Several case reports have described BP to be induced or exacerbated by exposure to ultraviolet light in the form of radiation therapy, psoralen with ultraviolet A (PUVA) or UVB therapy, and from local trauma from thermal or electrical burns, however none of these potential triggers have been explored in a case control study (29–38).

The pathogenic mechanism through which external agents could initiate or exacerbate PV and BP are not yet understood. Environmental factors may induce biological processes in the skin which lead to the exposure of self-antigens and the production of autoantibodies. This may be mediated by cytokine dysregulation or molecular mimicry leading to the development of humoral autoimmunity (39, 40). These processes underlie findings of epidemiological studies comparing environmental exposures between those with and without AIBD. Therefore, our aim was to investigate the associations of PV and BP with environmental and occupational risk factors previously reported in the literature.

## Materials and methods

Ethics approval for this study was obtained from the South Eastern Sydney Local Health District Human Research and Ethics Committee (number STG/08/66).

Case subjects were patients recruited from an academic dermatology clinic specialising in blistering disease. Inclusion criteria for patients with PV or BP were as follows: age ≥ 18 years, a diagnosis of PV or BP based on clinical, histopathological, and immunohistologic criteria. Interviews with patients were conducted during their scheduled appointments or over the telephone. The Australian Electoral Commission provided a list of 50 controls per case that were matched for age (± 2 years), birth gender, and residential location (within 10 km) through electoral records (41). Controls were first screened by name to match for ethnicity as closely as possible. The names and addresses of the controls were searched on the White Pages search engine (a public database) to obtain their telephone numbers. Control subjects were recruited by postal letter and phone call and those who agreed were interviewed for approximately 30 min over the telephone by the same clinicians who interviewed the cases. We attempted to recruit four to five controls per case however this was not possible for all cases. The medium number of cases per control recruited for BP was 3 (range 1–4) and the medium number of cases per control for PV was 3 (range 1–7) (Table 1).

**Table 1:** Number of matched controls per case.

	Bullous pemphigoid	Pemphigus vulgaris
Number of controls	Occurrences	Occurrences
1	2	3
2	7	4
3	11	15
4	9	1
5	0	1
6	0	0
7	0	1

Informed consent was obtained from all subjects prior to participation in the case–control study.

**Figure 1:**
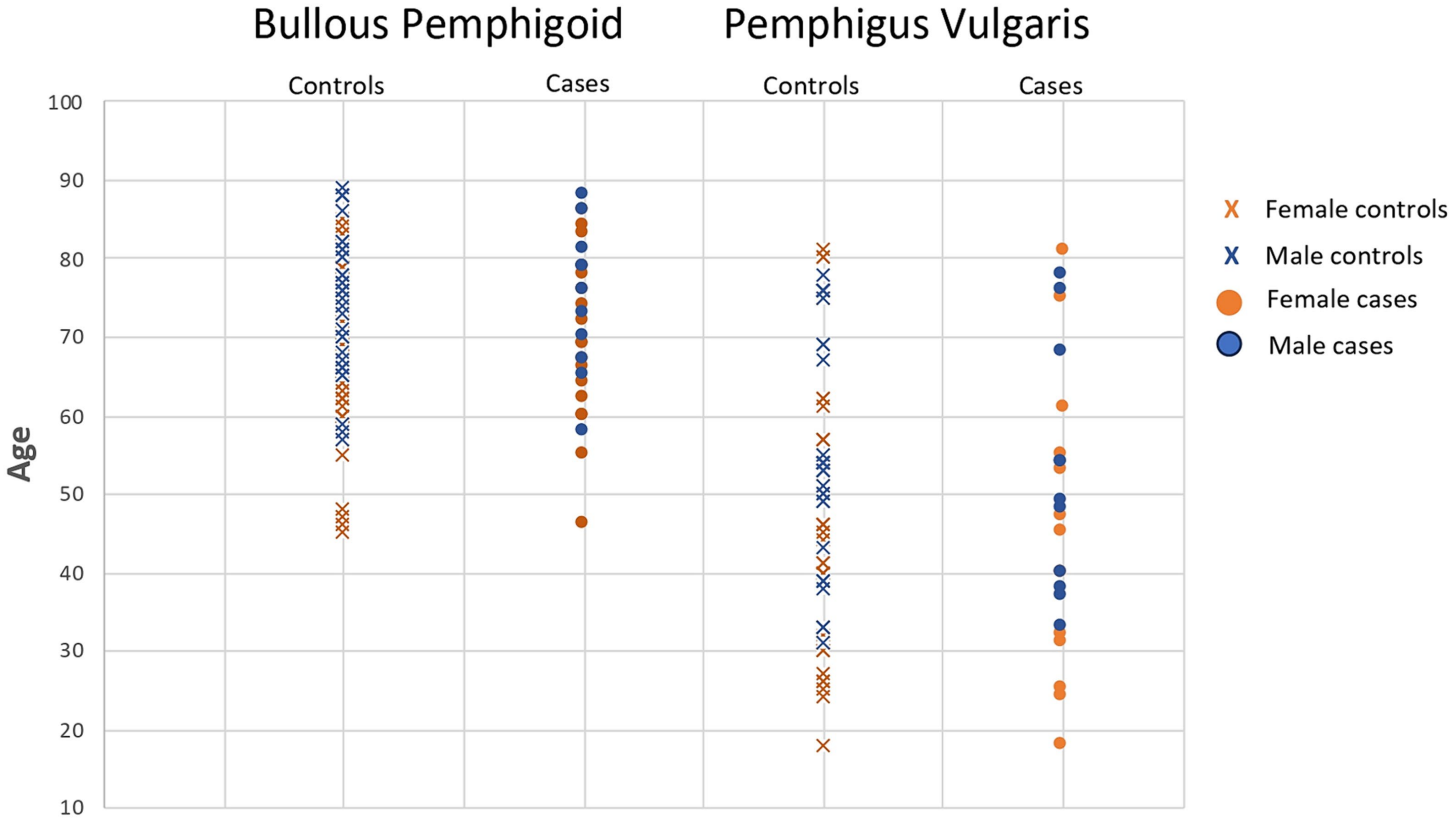
Bullous pemphigoid and pemphigus vulgaris cases and matched controls demonstrating age and gender matching for each case.

**Figure 2:**
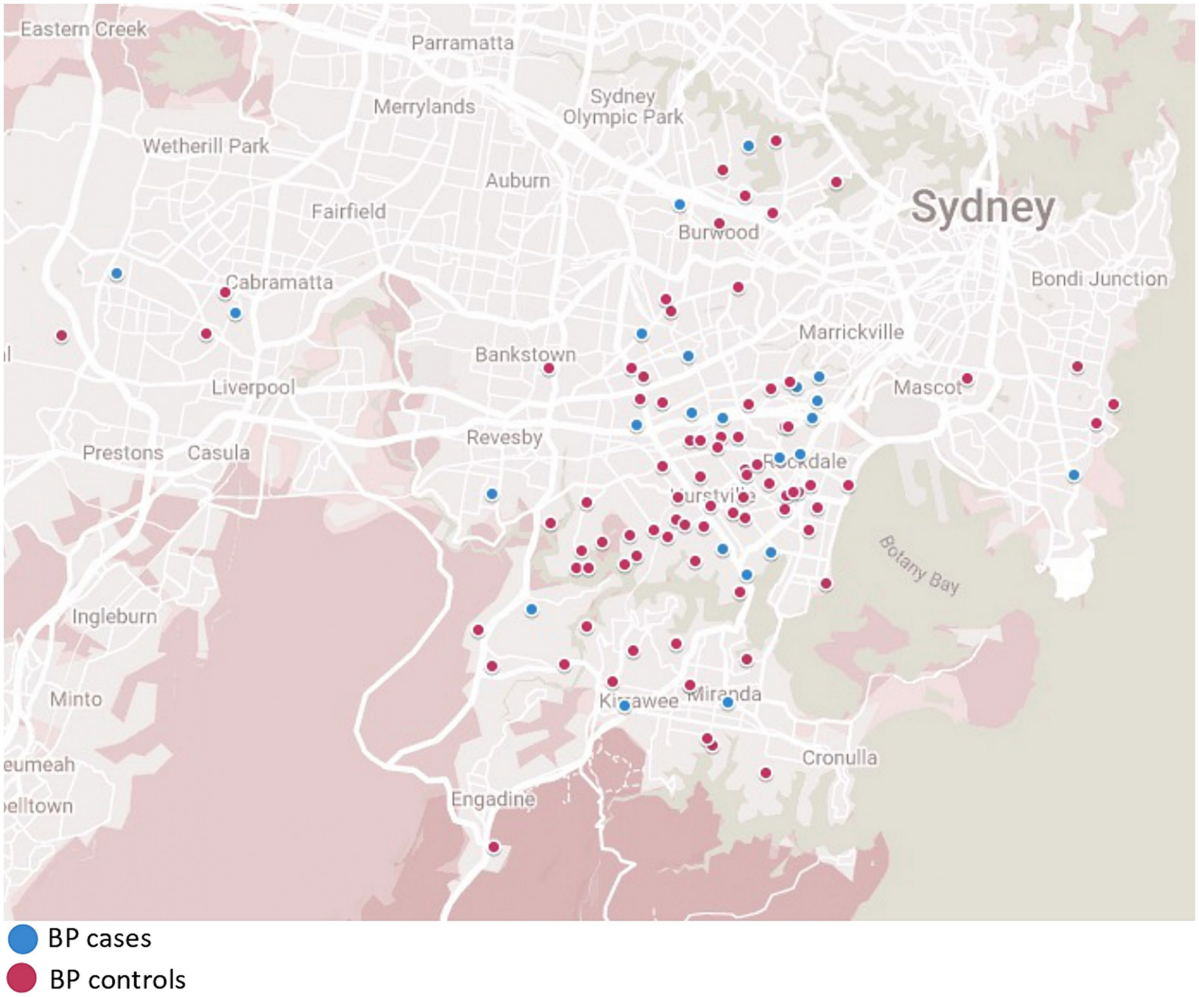
Bullous pemphigoid cases and matched controls demonstrating location matching of each patient.

**Figure 3:**
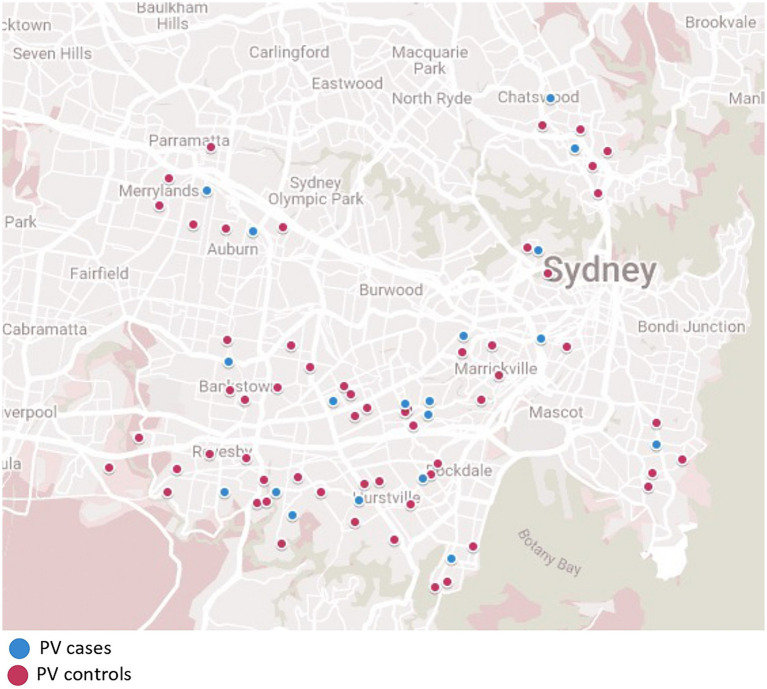
Pemphigus vulgaris cases and matched control demonstrating location matching of each patient.

A total of 25 PV patients (11 males, 14 females) and 72 control subjects (32 males, 40 females), and 29 BP patients (11 males, 18 females) and 84 control subjects (35 males, 49 females) were enrolled in the study (Figures 1–3). Data were collected using a structured questionnaire where cases were asked to reflect on their ‘usual lifestyle habits’ for the 12 months preceding disease onset and controls asked to reflect their usual lifestyle habits over the preceding 12 months. A number of distractor questions were included, thought not to be relevant to onset of either diseases.

Information included demographic factors, smoking history, supplement intake, immunisation history, alcohol intake, dietary factors, travel history, use of personal hygiene and household cleaning products, and occupational factors including the duration and mode of exposure (direct contact or vapour/fumes) to pesticides, organic product, heavy metals and other substances. Information on the daily duration of sunlight exposure and history of sunburns was collected. The use of hormonal medications and supplements, previous pregnancies, and subjective levels of emotional stress in the 12 months prior to diagnosis were also recorded (Supplementary material S1). The questionnaire given to case subjects included an additional section with questions regarding disease onset and duration, the distribution of lesions, previous treatments and hospitalisations, and current disease activity (active or in remission).

SPSS software was used to calculate chi-square and conditional logistic regression. The odds ratio (OR) and 95% confidence interval (CI) for each variable were calculated. Statistical significance was defined as *p* < 0.05.

## Results

### Pemphigus vulgaris

In total 25 PV patients and 72 control subjects matched for age, birth gender, residential location and in most cases, ethniciticy, were enrolled in the study (Table 2). Of the 25 PV patients there were 11 males (42%) and 14 females (58%). The median age at diagnosis was 47 years with a range of 18 years to 81 years. The largest proportion of patients were Caucasian (48%). The remaining were of Middle Eastern (24%), Asian (24%) and South Asian (4%) descent. Active disease was present in 8 patients (32%) 17 patients (68%) were in remission. Of the 12 patients (48%) who had been previously hospitalised due to their condition, 10 patients reported 1–2 hospital admissions in total and two patients reported 3–5 hospital admissions in total. Overall, PV patients reported an average of 2 new blisters per month.

**Table 2:** Distribution of matching factors between cases and controls.

		Bullous pemphigoid		Pemphigus vulgaris	
		Cases	Controls	Cases	Controls
Age	<60	3	12	19	53
	60–70	10	26	2	6
	70–80	10	29	3	10
	>80	6	17	1	3
Ethnicity	Caucasian	24	78	11	55
	Asian	4	5	8	12
	Middle Easter	1	1	6	5
Sex	Female	18	49	14	40
	Male	11	35	11	32

Dietary factors including the daily consumption of leeks (OR = 3.6; *p* = 0.025), mustard oil (OR = 4.4; *p* = 0.049), alcohol (*p* = 0.039), and tomatoes (OR = 4.735; *p* = 0.032) were significantly associated with pemphigus (Table 3). The daily use of supplementary medications including multivitamins (*p* = 0.009) and calcium (*p* < 0.001) were also found to be significant. High levels of self-reported emotional stress (categorised as “moderate”, “severe”, or “extreme”) in the 12 months leading up to diagnosis was a significant risk factor for pemphigus as was the number of lifetime sunburns (*p* = 0.019).

**Table 3:** Significant and marginally insignificant factors for pemphigus vulgaris.

Pemphigus vulgaris	
Factor	*p* Value
Previous travel to rural areas of Brazil/Columbia	0.088
Digoxin	0.088
DPP4-I	0.088
Daily tea consumption	0.071
Smoking	0.71
Calcium channel blocker	0.063
Daily celery consumption	0.062
Daily mustard oil consumption	0.049
Organic product vapour/fumes	0.045
Daily hot chocolate consumption	0.04
Daily EtOH consumption	0.039
Daily consumption of tomatoes	0.032
Daily consumption of leeks	0.025
Lifetime sunburns	0.019
Daily consumption of multivitamins	0.009
Alternative medications	0.001
Daily calcium supplement	0.001
Mental stress	0.001
Lime cleaning product	0.001

Refer to Supplementary material S1 for full list of questions and definitions of alternative medications.

Occupational exposure to organic products such as grass clippings, crops, compost, or animal manure (*p* = 0.045) and the use of lime household cleaning products (*p* < 0.001) were significantly associated with pemphigus patients whilst smoking was not associated (*p* = 0.944).

### Bullous pemphigoid

Data from 29 BP patients and 84 control subjects (35 males, 49 females) were enrolled in this case–control study. Of the 29 BP patients, 11 (37.9%) were male and 18 (62.1%) were female and the control group comprised 35 males (41.6%) and 49 females (58.3%). The median age at diagnosis was 72 years with a range of 46 years to 88 years. The largest proportion of patients in the BP group was Caucasian (79.3%) and the remainder of the patients were of Middle Eastern (3.4%), Asian (10.3%), South Asian (3.4%) and South American (3.4%) descent. Eight patients (27.6%) had active disease and 21 (72.4%) were in remission. Of the 7 patients (24.1%) who had been previously hospitalised due to their condition, 6 patients had been admitted 1–2 times and only 1 patient who had been admitted 3–5 times. Flares of new blisters every month was reported in a total of 13 patients (44.8%) and there was an average of 4.2 new blisters per month reported in this group overall.

The daily consumption of green or herbal tea (OR = 3.7; *p* = 0.004) was found to increase the likelihood of developing BP (Table 4). The use of supplementary medications such as fish oil (*p* < 0.001), calcium (*p* < 0.001), multivitamins (*p* = 0.043), and glucosamine (*p* = 0.046) were also found to be associated with BP. Other significant environmental exposures included mental stress (*p* = 0.007) and the use of lime as a household cleaning product (*p* < 0.001).

**Table 4:** Significant and marginally insignificant factors for bullous pemphigoid.

Bullous pemphigoid	
Factor	*p* Value
Lifetime sunburns	0.106
Exposure to heavy metals	0.098
Celery	0.094
Digoxin	0.088
DPP4-I medication	0.088
Daily echinacae	0.087
Daily hot chocolate	0.072
Smoking	0.682
Calcium channel blocker	0.063
Tomatoes	0.061
Daily glucosamine	0.046
Daily multivitamins	0.043
Mental stress	0.007
Daily green/herbal tea	0.004
Daily alternative medication use	0.001
Daily fish oil supplement	0.001
Daily calcium supplement	0.001
Lime cleaning product	0.001

Refer to Supplementary material S1 for full list of questions and definitions of alternative medications.

Occupational factors including exposure to pesticides, heavy metals, organic products, petroleum, general waste, asbestos, and latex were not statistically significant.

## Discussion

This is the first study to conduct detailed one-on-one questionnaires, including distractors, of PV and BP patients with three to four very well matched controls per case. Various environmental factors were investigated as suggested from prior studies undertaken.

### Dietary factors

Compounds such as phenols, thiols, and tannins (polyphenolic compounds) which are found in many of the causative medications associated with pemphigus are also present in high concentrations in a number of foods (42, 43). An experimental study has found that allyl compounds, found in garlic, leek, and onion, can provoke acantholysis in human skin cultured *in vitro*, with the acantholytic effect being most prominent in pemphigus prone tissue from an HLA-DR4+ donor, an HLA class II gene with a known association with pemphigus vulgaris (44). This suggests that histocompatability antigens can make the skin biochemically more *susceptible* to exogenous acantholytic factors. Tannic acid added to normal skin culture *in vitro* has also been shown to cause acantholytic changes (45).

Clinically, Ruocco et al. reported a case of 49-year-old male who developed pemphigus after consuming a large quantity of garlic. Without any conventional treatment the pemphigus cleared after a few months whilst on a garlic-free diet, however the pemphigus quickly returned after unintentionally consuming a strongly garlic-spiced meal (14). Foods high in isothiocyanate-producing glucosides (mustard seeds, and mustard oil), phenols (mangoes, black pepper, and nuts) and tannins (red chili pepper, tea, red wine, cherry, raspberry, cranberry, and blackberry) have also been linked with pemphigus (42).

Our study found significant associations (*p* < 0.05) between pemphigus and the daily consumption of leeks (*p* = 0.025), tomatoes (*p* = 0.032), mustard oil (*p* = 0.049), hot chocolate drinks (*p* = 0.04), and alcohol (*p* = 0.039). Marginally insignificant associations (*p* = 0.05–0.10) were also found with celery (*p* = 0.062), and green or herbal tea (*p* = 0.071).

A number of supplements were found to be associated with BP including glucosamine, calcium, multivitamins and fish oil. Dietary factors and the use of supplements are yet to be associated with BP in the literature to date. The novel findings of our study suggest the need for further research in this area.

### Mental stress

Our study found that moderate to extreme levels of stress in the 12 months prior to diagnosis significantly increased the likelihood of both PV (*p* < 0.001) and BP (*p* = 0.007).

There is a known association between the immune system and the nervous system, and epidemiological studies have identified emotional stress as a precipitating factor in the occurrence of many autoimmune diseases and the exacerbation of symptoms (46). The role of psychogenic immunomodulation in the pathogenesis of bullous disorders continues to be an emerging area of research.

Several case studies and reports have identified emotional stress as a precipitating factor in the development and exacerbation of pemphigus. In a study of 13 consecutive pemphigus patients, Cremniter et al. reported the presence of a life stressor in the year preceding diagnosis in 12 patients (47). In five cases the event was classified as traumatic, i.e., distressing beyond the range of usual human experience, as defined by the Diagnostic and Statistical Manual of Mental health Disorders.

In a study of 10 PV patients, the four patients with poor disease outcomes had elevated initial serum levels of TNF-*α* compared to those with good disease outcomes, 3 of whom reported severe emotional stress in the month leading up to disease exacerbation (48). This suggests that pre-treatment serum TNF-α levels may be an important prognostic indicator for pemphigus in correlation with stress levels.

Interestingly no studies have explored a link between psychosocial stressors and BP. This a perplexing gap in the literature considering the interconnected nature of the skin, nervous system, and immune system, which has frequently been brought to light in the context of BP’s association with neurological disease (49).

Psychosocial stress can induce neurogenic inflammation where neuroendocrine hormones alter or amplify cytokine production, resulting in immune dysregulation and possibly autoimmunity (50). It is also possible that stress interacts with other known risk factors such as altering the immune response to latent herpesvirus which has been linked to both PV and BP (51). This raises the question of whether stress interacts with other environmental factors to disrupt tolerance to self-tissues initiating an autoimmune event. Recall bias and the subjective nature of self-reported emotional stress may represent limitations to the validity of these findings.

### Ultraviolet exposure

Phototherapy (psoralen, UVB, and UVA) for the treatment of other dermatological conditions, such as psoriasis and mycosis fungoides, has been documented as a likely trigger for the development of BP. In these cases, BP lesions typically appear on pre-existing psoriatic lesions suggesting that phototherapy could trigger antigenic changes in the skin (52, 53).

The pathogenic mechanism through which external agents could provoke or exacerbate BP are not yet understood; however, a postulated theory is that in predisposed individuals with low titres of circulating autoantibodies tissue damage leads to recruitment of complement, inflammatory cells and circulating antibodies resulting in the formation of bullae (54).

Our study found a higher number of lifetime sunburns in PV patients than in controls (*p* = 0.019) however no such association was found with BP. A larger study with greater statistical power may be needed to affirm this association.

### Occupational exposures

Occupational exposures comprise a broad spectrum of potential chemicals and agents implicated in the pathogenesis of pemphigus in both case reports and case–control studies. Organophosphate pesticides block the nicotinic acetylcholinesterase channel, leading to the accumulation of acetylcholine and subsequent downregulation of nicotinic and muscarinic receptors (55). As acetylcholine receptors (AChRs) on keratinocytes function to regulate expression of desmogleins, chronic downregulation of AChRs may compromise cell–cell adhesion and induce blister formation (49). Active radicals of chemical compounds or their metabolites such as heavy metals and waste products may facilitate autoimmunity by altering desmosomal proteins in the skin or by interacting with immune mediators to activate B- and T-lymphocytes (56).

A multi-centre study by Brenner et al. found that exposure to pesticides and occupational exposure to metal vapours was significantly associated with an increased risk of PV (57). Valikhani et al. from Iran also reported that PV patients were approximately 3 times more likely to have been exposed to occupational pesticides than controls (52, 58).

Our study found that PV and BP were associated with increased exposure to lime cleaning products (*p* = 0.001) when compared to controls, however a statistically significant relationship was not found for pesticides, heavy metals, organic products and other substances including petroleum, general waste, asbestos, and latex. PV was found to be associated with exposure to organic products (grass clippings, crops, compost, or animal manure) in vapour form (*p* = 0.045) and the use of household lime cleaning products.

### Smoking

Several studies have reported a lower percentage of current or former smokers in pemphigus patients compared to individuals without pemphigus (6, 53, 54, 59).

Nicotine has been found to interact with nicotinic AChRs on keratinocytes to promote cell–cell adherence, prevent acantholysis and stimulate the healing of erosions (54, 60). Due to smoking being less common amongst female patients with pemphigus in other studies, it is postulated that smoking exerts anti-oestrogenic effects which could be protective against pemphigus, in a similar way to the inverse relationship between endometrial cancer and smoking.

There is particular interest in the potential of cholinergic drugs as a novel therapeutic approach to pemphigus lesions (61). Our study did not find an association between PV (*p* = 0.71) or BP (*p* = 0.68) and smoking.

## Limitations

Although control subjects were matched for age, sex, and residential location, it was not possible to match for patient ethnicity in every case due to ethical limitations. Ethnicity represents genetic predisposition to AIBD. We attempted to overcome this by choosing to interview controls whose family names were from the same ethnic group. This study was also susceptible to recall bias as patients were more likely to have scrutinised potential triggers to their disease and positively respond to questions regarding previous exposures. The presence of unequal cluster size present a statistical limitation in this study. Oversampling minority classes would lead to decreased generalisability and undersampling majority classes would lead to weaker statistical power. Due to the small sample size in this study weighting was also not used as it would have lead to overfitting. The small sample size and class imbalance are both statistical limitations in this study, therefore findings from this study are hypothesis generating only.

The use of a validated questionnaire including standardised instruments for measuring subjective parameters, such as stress, would be beneficial in future studies. Information and recall bias related to under- or over-reporting of food intake was also identified as a limitation in the dietary component of the questionnaire. The retrospective nature of our study was another limitation.

## Data Availability

The raw data supporting the conclusions of this article will be made available by the authors, without undue reservation.
