# Supplementary material for: Environmental triggers of pemphigus vulgaris and bullous pemphigoid: a case control study
Source: Front Med (Lausanne). 2024 Oct 22;11:1441369. doi: 10.3389/fmed.2024.1441369 (PMC11537152; doi:10.3389/fmed.2024.1441369)
Supplement: Supplementary file 1 [file Data_Sheet_1.pdf]

STRUCTURED QUESTIONNAIRE FOR ENVIRONMENTAL/OCCUPATIONAL TRIGGERS FOR PEMPHIGUS AND BULLOUS PEMPHIGOID

- What is your Date of Birth (DD/MM/YY)?
- Sex (M/F)?
- What is your ethnic background? Please tick:

Australian Indigenous/Torres Strait Islander		South Asian (Bangladesh/India/Pakistani/Sri Lankan)	
African		Caucasian	
Hispanic/Latino/South America		Pacific Islander/Polynesian	
Asian		African –American	

If other – please specify:

- Are you of Jewish descent? Y/N

If yes, are you of Ashkenazi descent? (Y/N)

- Are you of Muslim descent?

If yes, are you of:

  - Suni descent:
  - Shiite descent:
  - Other: Please specify

- What is your current Occupation/Job (if applicable)?

A. SMOKING

a.1 Do you smoke or have you smoked in the past? (Y/N)
If no, please proceed to section B

If yes,
a.2 (i) How old were you when you started smoking?

a.2 (ii) Do you smoke any of the following?

Cigars		Marijuana	
Pipe Smoking			

a.3 How many years have you smoked for (please tick)?

0-5 years		15-25 years	
6-10 years		>25 years	
11-15 years			

a.4 How many cigarettes do you smoke on an average daily basis?

0-5		16-25	
6-10		26-50	
11-15		>50	

Questionnaire designed by Dedee Murrell & Supriya Venugopal; licensed to the Australian Blistering Diseases Foundation. Requires permission to be used.

B. OCCUPATIONAL EXPOSURES

b.1 Please list your occupational (work) history from current job to first job:

- Current job (no of years worked):

- Previous jobs(no of years worked):

- Casual jobs

b.2 Do you believe that your current or previous jobs played a role in developing or triggering skin diseases (Y/N)

b.3 Are you exposed to pesticides as part of your current or previous job? (Y/N)

If yes:

- For how many years do/did you use pesticides)?

0-1 years		6-15 years	
1-5 years		>15 years	

- What type of pesticides were you exposed to? (please tick)

DDT		Dioxins	
PCBs		Diazinon	
Chlordane		Chlorpyrifos (Dursban)	
PCP		Other	

If you answered other – what was the name of the pesticide(s) you were exposed (if possible):

- How often do (did) you use pesticides at work (frequency): (please tick)

Daily		Fortnightly	
daily to weekly		Monthly	
Weekly		less than monthly	

- Do you wear personal protective equipment (PPE) when handling pesticides (Y/N)?
  If yes – please specify:

Rarely		Mostly	
Sometimes		Always	

b.4 Are you exposed to the following heavy metals as part of your current or previous job? (please tick)

Cadmium		Mercury	
Nickel		Beryllium	
Lead		Arsenic	
Chromium		Iron	

If yes:

- How were you exposed to this metal(s)?

Direct contact		Vapour/fumes	

- When and/or for how many years were you exposed to these metals?

0-1 years		6-15 years	
1-5 years		>15 years	

- Did you wear personal protective equipment when handling these metals?
  If yes please specify:

Rarely		Mostly	
Sometimes		Always	

b.5 Have you been exposed to the following organic products as part of your current or previous occupation/s? (please tick)

Grass clippings		Compost	
Crops		Animal manure	

If yes:

- How were you exposed to these substances?

Direct contact		Vapour/fumes	

- When and how for how many years were you exposed to these substances?

0-1 years		6-15 years	
1-5 years		>15 years	

- Did you wear personal protective equipment when handling these substances?
  (if yes please specify)

Rarely		Mostly	
Sometimes		Always	

b.6 Have you been exposed to the following substances as part of your current or previous occupations? (please tick)

Grease or grease traps		General rubbish or waste	
Oil		Asbestos	
Petroleum		Latex	

If yes:

- How were you exposed to these substances?

Direct contact		Vapour/fumes	

- How many years were you exposed to these substances?

0-1 years		6-15 years	
1-5 years		>15 years	

Questionnaire designed by Dedee Murrell & Supriya Venugopal; licensed to the Australian Blistering Diseases Foundation. Requires permission to be used.

- Did you wear personal protective equipment when handling these substances? If yes – please specify:

Rarely		Mostly	
Sometimes		Always	

b.7 If you had pre-existing skin disease, do you believe your current or previous occupation exacerbate or worsen your symptoms?

Yes		No	

Questionnaire designed by Dedee Murrell & Supriya Venugopal; licensed to the Australian Blistering Diseases Foundation. Requires permission to be used.

C. OTHER ENVIRONMENTAL TRIGGERS

c.1 Have you travelled overseas to the following regions?

Middle East		South America	
Africa		Europe	

If yes,

- Which country?
- When?

If answered: Brazil or Columbia – please proceed to c.2
All others proceed to c.3

c.2 Brazil/Columbia:

- Did you travel to rural areas of Brazil or Columbia (Y/N)
- Were you exposed to black fly insects (Diptera: Simulium) whilst in this country? (Y/N)

c.3 Does your diet contain daily consumption of any one of the following foodstuffs: (please tick)

Garlic		Artifical sweeteners	
Leeks		Cinnamon spices	
Mustard seeds		Mangoes	
Mustard oil		Nuts with shells	
Celery		Tomatoes	

c.4
Do you have daily consumption of the following hot drinks? (please tick)

Coffee		Green/Herbal Tea	
Tea (traditional)		Hot chocolate	

If yes, how many times a day would you drink?

1		4	
2		>4	
3			

Do you have daily consumption of the following cold drinks (please tick)

Colas		Iced coffee	
Diet Colas		Other soft drinks	
Red Bull or V or similar		Iced tea	

If yes, how many times a day would you drink?

1		4	
2		>4	
3			

Questionnaire designed by Dedee Murrell & Supriya Venugopal; licensed to the Australian Blistering Diseases Foundation. Requires permission to be used.

c.5 What regular medications are you currently taking?

c.6 Are you currently on the following hormonal medications or supplements:

Oral contraceptive pill		HRT	
Estrogen		None	
Testosterone		Other (please specify)	

HRT = Hormone replacement therapy

c.7 If you are a female, have you been pregnant before? (Y/N)

c.8 Are you taking any alternative medicines or vitamins (please tick)

Multivitamins		Algae Spirulina Platenesis	
Glucosamine		Gingko Biloba	
Fish Oil		Calcium supplements	
Echinacae		Other	

If you answered other, please indicate what type of vitamin/supplement:

c.9 Have you been vaccinated for the following diseases? Please tick and indicate when if possible

Rabies		Cervical cancer vaccine	
Yellow Fever		Tetanus (when was last known booster?):	
Tuberculosis		Diptheria	
Anthrax		Heptatitis B	

c.10 How often do you drink alcohol? (please tick)

Never		Weekly	
Less than once monthly		Twice weekly	
Monthly		Daily	
Fortnightly			

If you drink alcohol, how many drinks do you have when you do drink? (please tick)

1		6-10	
2		>10	
3-5			

c.11 How much exposure to sunlight do you have on an average day? (please tick)

Questionnaire designed by Dedee Murrell & Supriya Venugopal; licensed to the Australian Blistering Diseases Foundation. Requires permission to be used.

Less than 30 min		Between 5 and 8 hours	
Between 30 min and 1 hour		Between 8 and 12 hours	
Between 1 and 2 hours		Greater than 12 hours	
Between 2 and 5 hours			

c.12 When was the last time you had a sunburn?

- How many episodes of lifetime sunburn episodes have you had?

None		11-15	
Less than 3		>15	
3-5		Every year I get several sunburns	
6-10		I can't go outside without getting sunburn	

c.13 Were you been exposed to high levels of mental stress in the past 12 months? (Y/N)
If yes, please tick the following stressful event and indicate when this occurred?

Loss of job		Divorce/separation	
Loss of spouse/partner		Migration	
Loss of close relative/friend		Workplace stress	
Financial difficulty		Other	

If you ticked other – please specify:

How would you describe the level of stress you were under? (please tick)

Extreme – needed hospitalisation		Mild	
Severe – needed medications/medical help		Minor	
Moderate			

c.14 Do you engage in the following hobbies?

Gardening		Tinkering with cars	
Fishing		Sports	

If yes to sports: what type of sport do you play?

If yes to any of the above, how many hours per week do you engage in the above?

Questionnaire designed by Dedee Murrell & Supriya Venugopal; licensed to the Australian Blistering Diseases Foundation. Requires permission to be used.

0-1		>10-15	
>1-2		>15-25	
>2-5		>25	
>5-10			

c.15 Do you clean your house yourself? (Y/N)
if yes:

- Do you wear gloves? (please tick)

Never		Occasionally		Often		Always	

- How many hours per week do you clean your house?

0-1		>10-15	
>1-2		>15-25	
>2-5		>25	
>5-10			

- What type of products do you use?

Bleach		Lime	
Degreasants		Household Pesticides	
Soap		Dish washing Detergents	
Commercial household cleaners (e.g Mr Muscle)		Other	

If you ticked other, please specify

c.16

- Do you usually take showers or baths? Or both equally?

- How often to do you take a shower/bath?

Less than every two days		Twice daily	
Every two days		More than twice daily	
Daily			

- If bath, list what you usually use in the bath?
- Do you use hair dye?
  - If so: How long have you used hair dye?

- What types of shampoo do you use?

Questionnaire designed by Dedee Murrell & Supriya Venugopal; licensed to the Australian Blistering Diseases Foundation. Requires permission to be used.

Commercial shampoos		Shampoo plus conditioner in one	
Tar based shampoos		Oil	
Medicated shampoos		Other	

If other – please specify

c.17 How often do you brush your teeth?

Less than every two days		Twice daily	
Every two days		Thrice daily	
Daily		More than 3 times/Day	

What kind of toothpaste do you use?

c.18 Have you had any dental work done please tick?

Fillings		Braces	
Root canal		Teeth whitening	
Crowned teeth		Other	
Major hospital based surgery (i.e. to the jaw)			

If you ticked other – please specify:

- If you ticked yes to fillings, what type of fillings do you have?

Gold		Glass	
Mercury + Silver		Resin	
Ceramic		Other	

If you ticked other please specify?

c.19

Do you use mouthwash regularly (Y/N)

Do you floss your teeth regularly (Y/N)

THANK YOU FOR PARTICIPATING IN THIS SURVEY AND CONTRIBUTING TO ONGOING BLISTERING DISEASE RESEARCH

Questionnaire designed by Dedee Murrell & Supriya Venugopal; licensed to the Australian Blistering Diseases Foundation. Requires permission to be used.
